# Comparison of physical behavior estimates from three different thigh-worn accelerometers brands: a proof-of-concept for the Prospective Physical Activity, Sitting, and Sleep consortium (ProPASS)

**DOI:** 10.1186/s12966-019-0835-0

**Published:** 2019-08-16

**Authors:** Patrick Crowley, Jørgen Skotte, Emmanuel Stamatakis, Mark Hamer, Mette Aadahl, Matthew L. Stevens, Vegar Rangul, Paul J. Mork, Andreas Holtermann

**Affiliations:** 1 The National Research Centre for the Work Environment, Copenhagen, Denmark; 2 School of Public Health, Charles Perkins Centre Prevention Research Collaboration, University of Sydney, Sydney, Australia; 3 School Sport Exercise, Health Sciences, Loughborough University, Loughborough, UK; 4 Center for Clinical Research and Prevention, Bispebjerg and Frederiksberg Hospital, Frederiksberg, Denmark; 5 HUNT Research Centre, Department of Public Health and Nursing, Faculty of Medicine, Norwegian University of Science and Technology (NTNU), Trondheim, Norway; 6 Department of Public Health and Nursing, Faculty of Medicine, Norwegian University of Science and Technology (NTNU), Trondheim, Norway; 7 Department of Sports Science and Clinical Biomechanics, University of Southern Denmark, Odense, Denmark

**Keywords:** Harmonization, Data pooling, Objective measurement, Tri-axial, Accelerometry, Health, Validation, Posture

## Abstract

**Background:**

Pooling data from thigh-worn accelerometers across multiple studies has great potential to advance evidence on the health benefits of physical activity. This requires harmonization of information on body postures, physical activity types, volumes and time patterns across different brands of devices. The aim of this study is to compare the physical behavior estimates provided by three different brands of thigh-worn accelerometers.

**Methods:**

Twenty participants volunteered for a 7-day free-living measurement. Three accelerometers - ActiGraph GT3X+, Axivity AX3 and ActivPAL Micro4 - were randomly placed in a vertical line on the midsection of the right thigh. Raw data from each accelerometer was processed and classified into 8 physical activities and postures using the Acti4 software. Absolute differences between estimates and the respective coefficient of variation (CV) were calculated.

**Results:**

We observed very minor differences between physical behavior estimates from three different accelerometer brands. When averaged over 24 h (1,440 min), the absolute difference (CV) between accelerometers were: 1.2 mins (0.001) for lying/sitting, 3.4 mins (0.02) for standing, 3.5 mins (0.06) for moving, 1.9 mins (0.03) for walking, 0.1 mins (0.19) for running, 1.2 mins (0.19) for stair climbing, 1.9 mins (0.07) for cycling. Moreover, there was an average absolute difference of 282 steps (0.03) per 24 h.

**Conclusions:**

Physical behaviors were classified with negligible difference between the accelerometer brands. These results support harmonization of data from different thigh-worn accelerometers across multiple cohorts when analyzed in an identical manner.

**Electronic supplementary material:**

The online version of this article (10.1186/s12966-019-0835-0) contains supplementary material, which is available to authorized users.

## Background

Accurate and accessible information quantifying daily physical behavior is vital for increasing public awareness and improving policy making related to physical activity and health. To attain this information, we require accurate estimates of how much time people spend in key physical behaviors, e.g. sitting, moving about, standing, walking, climbing stairs, cycling and running. These estimates can be obtained using thigh-worn accelerometers, which are capable of accurately identifying and distinguishing between key daily physical behaviors [1]. If the data from thigh-worn accelerometers could be harmonized and pooled across many cohorts, this would be considered the ‘state-of-the-art’ in physical activity and health evidence [2].

International consortia - like the International Children’s Accelerometry Database (ICAD) – have already established successful platforms for harmonizing and pooling accelerometer data [3] across multiple cohorts. However, such platforms have limitations. ICAD, for example, is limited to data from accelerometers placed on the hip [3], a placement which cannot simultaneously delineate the position of the lower limbs and trunk and therefore cannot provide the information required to accurately identify the aforementioned physical behaviors.

Although thigh-worn accelerometers have now been included in several large cohort studies such as HUNT4 [4], the BCS70 [5] and the Copenhagen City Heart Study [6], to date, no effort has been made to harmonize and pool thigh-worn accelerometry data across international cohorts. This is a goal of the Prospective Physical Activity, Sitting, and Sleep (ProPASS) consortium, which aims to provide a platform for research collaboration drawing together existing and future epidemiological studies that collect data using thigh-worn accelerometers [7]. In achieving this, ProPASS will act as a comprehensive and enduring source of information, with the potential to greatly enhance the understanding of daily physical behavior.

However, as these existing cohorts using thigh-worn accelerometers have used accelerometers from different brands (e.g. ActiGraph GT3x+, Axivity AX3 and ActivPAL Micro4), it is unclear whether these data can in fact be pooled and harmonized. Empirical evidence documenting the harmonization potential of thigh worn accelerometry data collected using different accelerometer brands is a fundamental requirement for the ProPASS initiative, and therefore, a novel methodology to assess this harmonization potential is required. This assessment could be achieved through the use of a single, validated software program to process the data collected by accelerometers from different brands, with a view to comparing the processed outcome. Therefore, the aim of this study is to compare estimates of key daily physical behaviors derived from three widely used thigh-worn accelerometer brands using the same validated software – Acti4 [1].

## Methods

We recruited participants by email circulated at the National Research Center for the Work Environment, Denmark. Potential participants were excluded if they reported any medical or physical constraint that would restrict the performance of the prescribed physical behaviors, were pregnant, or reported any skin problem that may be affected by the adhesive cover film of the accelerometers. All participants provided written informed consent. The Committee of Scientific Research Ethics for the Copenhagen Region provided ethical approval for the study (j.nr. 18,005,389).

### Accelerometer initialization

Accelerometers from three different manufacturers; ActiGraph GT3X+ (ActiGraph Ltd., Pensacola, Florida, US), Axivity AX3 (Axivity Ltd., Newcastle, UK), ActivPAL Micro4 (PAL technologies, Glasgow, Scotland) were used for data collection (for specifications see Additional file 1: Table A2). Each accelerometer was set to record in full power mode and at sampling frequencies of: Actigraph = 30 Hz, Axivity = 25 Hz and ActivPAL = 40 Hz respectively.

### Accelerometer calibration and placement

**Fig. 1:**
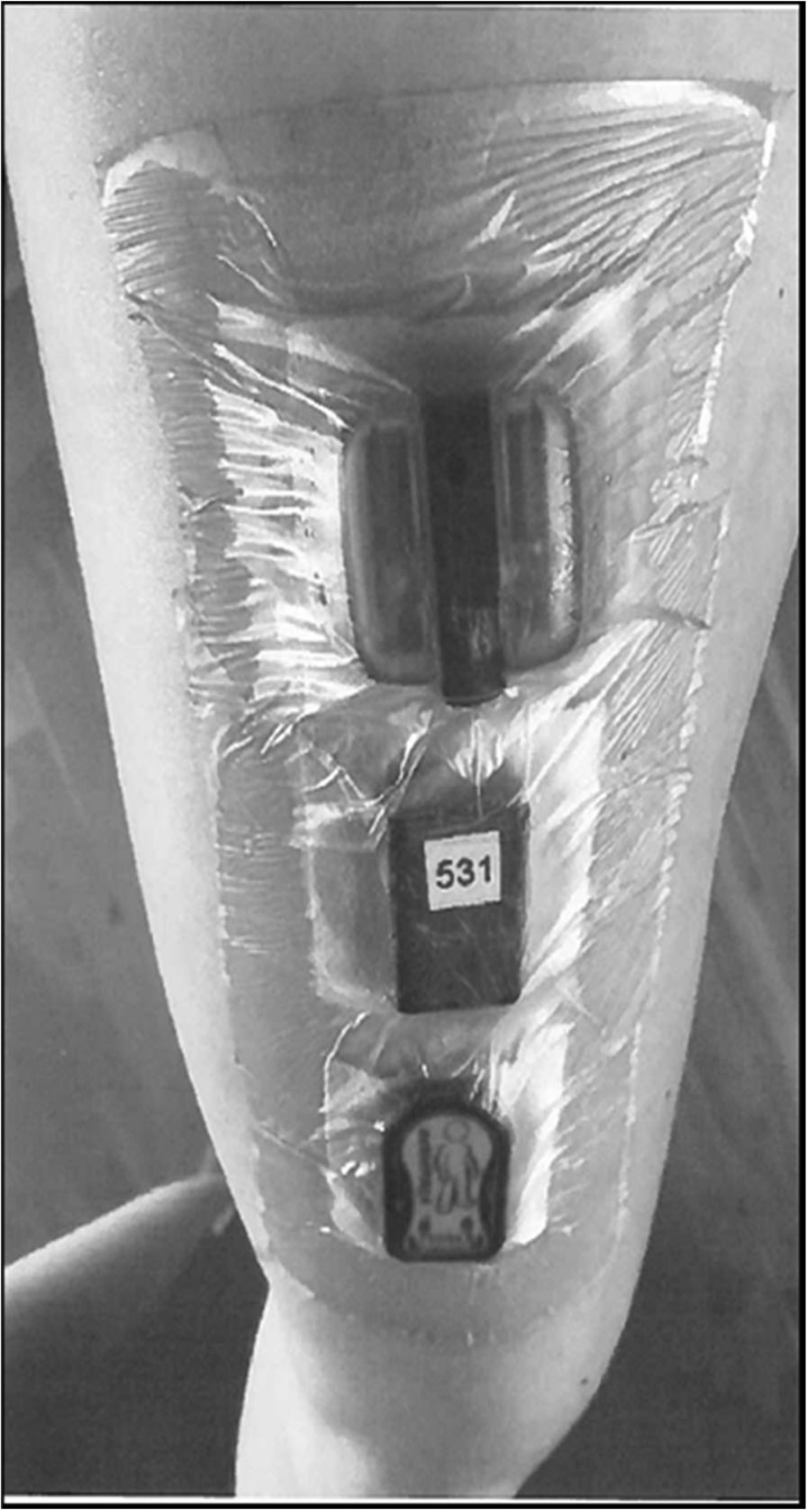
Illustration of accelerometer placement (top to bottom: Actigraph GT3X+, Axivity AX3, ActivPAL Micro) in a vertical line on the mid-section of the thigh. Accelerometers were placed approximately 2 cm apart and were attached directly to the skin using double sided tape. The order of the accelerometer placement was followed a randomized partial counterbalance design

All accelerometers were assessed for baseline measurement error. These measurements were obtained by aligning accelerometers - grouped according to manufacturer - flat along the inside of a transparent cube (10 cm × 10 cm × 4 cm) made of hard plastic. The cube was then placed on each of its six sides, for 5 s on each side. This process allowed the measurement error along each recording axis to be identified. Accelerometers were then sealed with cling-film, attached directly to the skin of the participant using double-sided adhesive tape (Hair-Set, 3 M Company, USA), and covered using an adhesive cover (Opsite Flexifix; Smith & Nephew plc, UK). The accelerometers (AX3, Micro, and GT3X+) were positioned on the right thigh, approximately midway between the anterior superior iliac spine and the patellar tendon (Fig. 1). The placement position of each accelerometer followed a randomized partial counterbalance design, while ensuring that each accelerometer had an equal number of recordings at the approximate midpoint of the thigh. In each case, the remaining two accelerometers were placed approximately 2 cm above and below this midpoint (Fig. 1).

### Measurement of physical behavior

Participants were asked to complete both a 15-min semi-standardized protocol and a 7-day free-living protocol. During the semi-standardized protocol, activities were performed in both an indoor and outdoor setting. Walking, stair climbing, sitting, and standing were assessed along a brightly lit indoor corridor, and adjoining stairwell. Running and cycling were performed outdoors in an adjacent carpark. The order and intensity of these activities were self-determined, and all semi-standardized measurements were recorded by an experimenter shadowing the participant with a handheld video camera (Garmin VIRB Pro Camera). A recording mode with a resolution of 1080p and a 1 frame-per-second precision was selected. Participants were instructed that they had 15- min to complete all activities and that each activity must be performed at least twice within this 15-min period.

Once the semi-standardized protocol was completed, the free-living protocol began. Participants were asked to wear the accelerometers over the next seven days, 24 h a day. A diary was provided allowing participants to log daily routines during measurement. The diary was formatted so that the participant could fill in the date of each measurement day, any experienced issues and eventual accelerometer removal. It was emphasized that participants should remove accelerometers at any sign of skin irritation; especially since the skin area covered in this study was quite large compared to the typical accelerometry measurement.

### Data processing

Proprietary software packages were used to initialize and download data from each respective accelerometer brand (Additional file 1: Table A2). Raw tri-axial accelerations (units in g) were extracted from each accelerometer and adapted to the file format compatible with Acti4 (.act4) [1]. For inclusion in analysis, data from all three accelerometers was required with discernible data from at least one complete 24-h period (midnight to midnight). Measurement periods between the semi-standardized session and midnight on the first day were excluded, as were periods between the last midnight time point and accelerometer removal, leaving a maximum of six days of recording for analysis.

### Classification of physical behavior

Physical behaviors were classified using Acti4, custom developed software – freely available upon agreement with the National Research Centre for the Working Environment, Copenhagen - that has demonstrated excellent sensitivity and specificity (93 to 100%) in both semi-standardized and free-living settings [1, 8, 9]. Acti4 uses a 2 s window with 50% overlap to separate physical behaviors into physical activity and postures types. This separation is done through a rule based algorithm, which uses the distributions of both accelerometer inclination and the maximal standard deviation of thigh accelerations [1]. This method allows the duration of measurement time spent in each of these physical behaviors to be calculated. The physical behaviors defined by Acti4 include; lying/sitting (the distinction between sitting and lying is only discernible with the addition of an accelerometer placed on the trunk), standing, moving about (upright movement which is neither classified as standing still or walking), walking, stair climbing, running, cycling, and stepping. The criteria for these classifications has been described previously [1, 10]. Acti4 derived the time spent in each physical behavior for the semi-standardized and free-living measurements, respectively.

## Statistical analysis

### Comparison between accelerometry-based estimates of physical behavior

The agreement between each accelerometer was assessed using Bland-Altman plots [11], coefficient of variation (CV), and the absolute SD (AbsSD) computed in minutes. First, we calculated the average SD between the estimate of physical behavior duration provided by each accelerometer for a given physical activity type or posture. Hereafter, we gave the term AbsSD to this value. To calculate the CV values, we divided this AbsSD by the mean of physical behavior duration for a given physical activity or posture. This combined mean was calculated as the mean physical behavior estimate across all three accelerometers. This was done for each participant, providing an AbsSD and CV value for each. Second, we compared the pairwise agreement in physical behavior estimates, for each accelerometer pair using Bland-Altman plots. To aid interpretation, averages for semi-standardized measurements were scaled up to a proportion of one hour. In the analysis of free-living measurements, averages were calculated as a 24-h average over the total number measurement days for each participant. Statistical analysis was conducted in SPSS (IBM Statistics Data Editor v.24) and Microsoft Excel (v.2010).

### Comparison between accelerometry-based estimates of physical behavior and video observation

Video observation of the semi-standardized measurement period was reviewed and annotated, second by second, separately by two researchers. That is to say 1 s of Acti4 classification was compared to 1 s of video observation. The definitions used for annotation can be found in Additional file 1: section E. Inter-rater agreement was calculated (Cohen’s Kappa) [12], as was the specificity and sensitivity of comparisons between each accelerometer-based physical behavior classification and the classification based on video observation. Sensitivity was defined as the proportion of classifications that are correctly classified as belonging to the physical activity type or posture of interest, in agreement with the criterion measure (video observation). Specificity was defined as the proportion of classifications that were correctly identified as not belonging to the physical activity type or posture of interest, in agreement with the criterion measure (video observation). Sensitivity and specificity are reported as a percentage. Agreement criterion for sensitivity and sensitivity were defined as slight (0.00–0.20), fair (0.21–0.40), moderate (0.41–0.60), substantial (0.61–0.80), and almost perfect (0.81–1.00) [13].

## Results

Following recruitment, 20 participants provided informed consent and were included in the study. Of these 20 participants, all completed the semi-standardized testing, but one did not complete the daily living measurement that followed. Therefore, the data from 19 participants is reported for the free-living measurements. Basic descriptive characteristics of participants are presented in Table 1. Eight participants reported removing the device before the 7 days were complete, of which, two participants reported skin irritation and one participant reported an accelerometer falling off. The reported skin irritation is likely due to the relatively large surface area covered by three accelerometers (Fig. 1), when compared to the typical placement of just a single accelerometer. There was a slight predominance of female participants and the average age of participants was 33 years.

**Table 1:** Descriptive statistics of all participants (*n* = 20)

	Mean ± 1SD or n
Age (years)	33 ± 12
Male/female	8/12
Height (cm)	173 ± 8
Weight (kg)	72 ± 13
Free-living measurements (days)	5 (Range: 2 to 6)

*SD* standard deviation

### Classification of physical behavior – semi-standardized protocol

Table 2 presents the agreement between accelerometry measurements gathered during the semi-standardized protocol and video observation of that protocol. Cohen’s kappa for inter-rater agreement for all physical behaviors measured during the semi-standardized protocol ranged from 0.89 to 0.99, except for the ‘moving about’ classification which produced kappa value of 0.16. The specificity of the physical behavior classifications provided by the Acti4 software ranged from 93% (moving about) to 100% (running and stair walking). The sensitivity was > 90% for classifying lying/sitting, walking, running, and cycling and somewhat lower for stand, move, and stair climbing.

**Table 2:** Agreement between Acti4 accelerometry-based classifications and video recording of the semi-standardized protocol (*n* = 20)

Accelerometer		Sit	Stand	Move	Walk	Run	Stairs	Cycle
Actigraph GT3x	Sensitivity (%)^(a)^	99	75	67	93	92	71	94
	Specificity (%)^(b)^	97	99	93	95	100	100	98
Axivity AX3	Sensitivity (%)^(a)^	96	73	63	91	95	78	93
	Specificity (%)^(b)^	97	98	94	94	100	100	99
ActivPAL Micro4	Sensitivity (%)^(a)^	99	74	55	93	94	72	94
	Specificity (%)^(b)^	97	99	93	95	100	100	98

Physical behaviors were recorded at 1 frame-per-second using a handheld video camera. Percentage of specificity and sensitivity provides a measure of the percentage of classification agreement between each accelerometer brand and video observation

^(a)^ Sensitivity is defined as the proportion of classifications that are correctly classified as the actual physical activity type or posture, in agreement with the criterion measure (video observation)

^(b)^ Specificity is defined as the proportion of classifications that were correctly identified as NOT belonging to the physical activity type or posture of interest, in agreement with the criterion measure (video observation)

### Classification of physical behavior – free-living protocol

Table 3 presents the average time spent in a given physical behavior during 24 h of free-living measurement. Average AbsSD (CV values in parentheses) values were 1.2 min (0.001) for lying/sitting, 3.4 min (0.02) for stationary standing, 3.5 min (0.06) for moving about, 1.9 min (0.03) for walking, 0.1 min (0.19) for running, 1.2 min (0.19) for stair climbing, and 1.9 min (0.07) for cycling. AbsSD for average steps per day was 282 steps with a CV of 0.03 (Table 3). Bland-Altman plots compared the mean physical behavior duration and the difference in the estimated duration provided by each accelerometer pair (see Additional file 1: sections B, C, & D). The systematic difference was minimal for all pairings, e.g., the largest bias present was between Actigraph GT3X+ and ActivPAL Micro4 for duration of standing time equating to 0.06 h (3.6 min) over 24 h. Relative to these small differences observed for other physical behavior estimates, a large difference was observed in the comparisons of step count - a difference of 323 steps per 24 h, when Actigraph GT3X+ count estimates were compared with Axivity AX3 count estimates, and a difference of 230 steps per 24 h when ActivPAL Micro4 estimates were compared to Axivity AX3 estimates.

**Table 3:** Physical behavior duration and step count per 24-h (1440 min) free-living (*n* = 19); mean ± 1SD

Accelerometer	Lie/Sit	Stand	Move	Walk	Run	Stairs	Cycle	Steps
	*(in minutes)*		*(in minutes)*					*(No. of steps)*
Actigraph GT3X+ ^(a)^	1063 ± 112	190 ± 82	66 ± 22	78 ± 21	3 ± 9	6 ± 3	33 ± 16	9920 ± 3097
ActivPAL Micro4 ^(a)^	1062 ± 113	193 ± 87	63 ± 20	78 ± 20	3 ± 9	5 ± 3	34 ± 17	9827 ± 2971
Axivity AX3 ^(a)^	1063 ± 113	192 ± 85	66 ± 20	76 ± 20	3 ± 9	6 ± 2	34 ± 16	9597 ± 2895
	Lie/Sit	Stand	Move	Walk	Run	Stairs	Cycle	Steps
AbsSD ^(b)^	1.2 ± 1.4	3.4 ± 3.2	3.5 ± 3.1	1.9 ± 1.5	0.1 ± 0.1	1.2 ± 1.1	1.9 ± 2.2	282 ± 276
CV ^(c)^	0.001	0.02	0.06	0.03	0.19	0.19	0.07	0.03

^(a)^Behavior classifications are based on those defined by Skotte et al. 2014 [1]. Step count was derived according to Ingebrigtsen et al. 2013 [10]. Values are computed from the daily average duration of free-living accelerometry measurements of up to a maximum of 6 days. Accelerometers from three different brands (Actigraph GT3+, Axivity AX3, and ActivPAL Micro4) were placed in a vertical line on the midsection of the thigh. The order of placement followed a randomized partial counterbalance design

^(b)^Mean SD is calculated as the *average* standard deviation in activity durations between all three devices, for each participant

^(c)^$\mathrm{CV}=\upsigma /\overline{\mathrm{A}}$; *where* *σ* = SD and $\overline{A}$ = the mean activity duration between all three devices for each participant

## Discussion

We observed minimal differences in the duration of the free-living physical behavior estimates between the three thigh-worn accelerometer brands, after data were analyzed in an identical manner. Furthermore, substantial to almost perfect agreement was found for almost all physical behaviors from the thigh-worn accelerometry data referencing frame-by-frame video annotation during measurement under semi-standardized conditions.

### Strengths and limitations

This study is strengthened by assessment of the most common accelerometers used in large cohorts around the world [4–6] – with added relevance to the implications regarding the stated aim of ProPASS. An additional strength of our protocol is the implementation of both semi-standardized and free-living measurements. This design provides the opportunity to verify the results observed in controlled conditions with those observed in more typical, every-day conditions. Moreover, measurement in the semi-standardized condition also allowed for further verification of accelerometry-based physical behavior classifications against video observation. Although the inability to perform verification against video observation during free-living measurements could be considered a limitation of this study, such observation was not feasible. Another potential limitation is the limited verification capacity of video observations for those activities with lower inter-rater agreement (i.e. moving about, stair walking, standing). Finally, our sample population were recruited from a relatively small, single workplace, and thereby provide limited heterogeneity in subject characteristics (e.g. age), which limits the generalization of the current findings.

### Implications of the study

Our findings provide empirical support for the potential to harmonize data across different thigh-worn accelerometer brands, when data are analyzed in an identical manner. Although we found minor differences in absolute duration and coefficient of variation (CV) estimates of the physical behaviors in a free-living setting between accelerometer brands, the negligible size of these differences indicate that they are unlikely to be of any practical consequence. Even the somewhat higher CV values identified for running and stair climbing are due to the much shorter mean durations of these physical activities and thus are also unlikely to be of any importance. This interpretation is supported by the Bland-Altman plots, in which most of the physical activity and postures types cluster around the group mean (See Additional file 1: Sections B to D).

The findings from free-living measurements are supported by the percentage classification agreement between thigh-worn accelerometry, as classified by Acti4, and the criterion measure (video observation) of physical behaviors measured in the semi-standardized setting. The specificity of the Acti4 classification was ≥93% for all physical behaviors. Sensitivity was also excellent for the lying/sitting, walking, running and cycling classifications; but somewhat lower for stand, move, and stair climbing. The lower sensitivity indicates a difficulty in establishing the ground truth or criterion measure for these categories during the video annotation. This may, in part, be due to the choice of a 1 frame-per-second video sampling rate, making it very difficult to discriminate between standing, moving about, and walking. These high values for specificity and sensitivity mean that the study not only provides “a proof of concept” for the harmonization of thigh-worn accelerometer data across multiple cohorts using a single validated software like Acti4, but also reaffirms the accuracy of these measurements.

### Future research

Because thigh-worn accelerometers can deliver information on key daily human physical behaviors, like sitting, standing, walking, stair climbing, cycling and running, we see a great potential in the ProPASS consortium forming a research collaboration platform for pooling cohorts with thigh-worn accelerometer data – opening many opportunities for future research. Harmonization and pooling of accelerometer data from large cohorts is an important next step to improve the evidence base on physical activities and health [2]. In the short-term, future research should aim to investigate the feasibility of retrospective harmonization of accelerometry-based variables derived from different accelerometer brands and processed using proprietary analysis software.

### Conclusions

We present a novel methodology for the comparison of physical behavior estimates provided by accelerometers from different brands. We found negligible differences in the duration of the free-living physical activities and postures between the three thigh-worn accelerometer brands. Our study gives empirical support for the ability to accurately harmonize data from different thigh-worn accelerometer brands when using the Acti4 software. Thus, this study provides “a proof of concept” for harmonizing thigh-worn accelerometer data across multiple cohorts.

## Additional file

Additional file 1: Supplementary data, figures & definitions. Sections A to E detail the average physical behavior durations during semi-standardized measurements, present Bland-Altman plots of between-accelerometeragreement and describe the definitions used for physical behavior classification during video annotation. (DOCX 1121 kb)

## Data Availability

Data can be made available upon reasonable request to the corresponding author.

## Abbreviations

AbsSD: Absolute standard deviation
CV: Coefficient of variation
ICAD: International Children’s Accelerometry Database
ProPASS: Prospective Physical Activity and Sitting and Sleep consortium
SD: Standard deviation
