# Supplementary material for: Comparison of physical behavior estimates from three different thigh-worn accelerometers brands: a proof-of-concept for the Prospective Physical Activity, Sitting, and Sleep consortium (ProPASS)
Source: Int J Behav Nutr Phys Act. 2019 Aug 16;16:65. doi: 10.1186/s12966-019-0835-0 (PMC6697962; doi:10.1186/s12966-019-0835-0)
Supplement: Supplementary file 1 — Supplementary data, figures & definitions. Sections A to E detail the average physical behavior durations during semi-standardized measurements, present Bland-Altman plots of between-accelerometeragreement and describe the definitions used for physical behavior classification during video annotation. (DOCX 1121 kb) [file 12966_2019_835_MOESM1_ESM.docx]

**Appendix**

**Section A**
Table A - Average physical behavior duration, scaled to 1 hour, from the semi-standardized protocol

Table A2 – Basic specification descriptors of each thigh-worn accelerometer used in the current study

**Section B**
Bland-Altman plots showing the agreement between Actigraph Gt3x and ActivPAL Micro4 measurements, collected during the free-living protocol

**-**Figure 1(a)

-Figure 1(b)

**Section C**
Bland-Altman plots showing the agreement between Actigraph Gt3x and Axivity AX3 measurements, collected during the free-living protocol

-Figure 2(a)

-Figure 2(b)

**Section D**

Bland-Altman plots showing the agreement between ActivPAL Micro4 and Axivity AX3 measurements, collected during the free-living protocol

-Figure 3(a)

-Figure 3(b)

**Section E**

Definitions used in the annotation of video observations

**Section A**

Table A **-** Average physical behavior duration and step count over 60 minutes (n = 20 participants); mean ± 1SD

|  | Sit | Stand | Move | Walk | Run | Stairs | Cycle | Steps |
| --- | --- | --- | --- | --- | --- | --- | --- | --- |
|  | *(in minutes)* | | *(in minutes)* | | | | | *(no. of steps)* |
| Actigraph GT3x ^(a)^ | 8 ± 3.9 | 12.3 ± 4.7 | 5± 1.5 | 16 ± 4.1 | 7 ± 2.6 | 4 ± 1.5 | 8 ± 2.4 | 992 ± 186 |
| ActivPAL Micro4 ^(a)^ | 8 ± 4.4 | 12.2 ± 4.3 | 5 ± 1.3 | 16 ±3.7 | 7 ± 2.5 | 4 ± 1.8 | 9 ± 3.1 | 980 ± 184 |
| Axivity AX3 ^(a)^ | 8 ± 5.3 | 11.9 ± 3.4 | 5 ± 1.6 | 16 ± 3.8 | 7 ± 2.6 | 4 ± 1.2 | 9 ± 2.7 | 972 ± 180 |
|  | Sit | Stand | Move | Walk | Run | Stairs | Cycle | Steps |
| AbsSD ^(b)^ | 0.1 ± 0.25 | 0.2 ± 0.26 | 0.2 ± 0.13 | 0.2 ± 0.13 | 0.1 ± 0.08 | 0.2 ± 0.17 | 0.20 ± 0.29 | 28 ± 21 steps |
| CV ^(c)^ | 0.05 | 0.04 | 0.16 | 0.04 | 0.04 | 0.18 | 0.07 | 0.03 |

(a) Activity classifications are based on those defined by Skotte et al. 2014 (1). Step count was derived according to Ingebrigtsen et al. 2013 (10).Values are computed from the average duration of semi-standardized accelerometry measurements. Accelerometers from three different brands (Actigraph Gt3x+, Axivity AX3, and ActivPAL Micro4) were placed in a vertical line on the midsection of the thigh. The order of placement was randomized and balanced. Proportion was calculated as: $scaled mean\boldsymbol{=}\frac{x}{17.350}*60$; where $x$ is the original mean and the mean measurement duration is 17.350 minutes

(b) Mean SD is calculated as the *average* standard deviation in activity durations between all three devices, for each participant.

(c) $CV=\sigma/\overline{A}$; *where* $\sigma$ = SD and $\overline{A}$ = the mean of activity duration between all three devices, for each participant

Table A2 – Technical features of the ActiGraph GT3X+, Axivity AX3, and ActivPAL Micro4 accelerometers

| Attribute | Actigraph GT3X+ | Axivity AX3 | ActivPAL Micro4 |
| --- | --- | --- | --- |
| Sensing unit | MEMS | MEMS | MEMS |
| Range | ±6g | ±2-16g | ±2-4g |
| Dimensions | 19 × 34 × 45mm | 7.6 × 23 × 32.5mm | 23.5 × 43 × 5mm |
| Default Sample  Frequency | 30 Hz | 100Hz | 20 Hz |
| Manufacturer recommended position | Wrist, waist, ankle, thigh | None specifically | Right mid-thigh |
| Orientation Sensitive | Yes (correctable if positioned upside down) | Yes (correctable if positioned upside down) | Yes (correctable if positioned upside down) |
| Raw file | .GT3x(.csv available) | .CWA (.csv available) | .PAL (.csv available) |
| Software | ActiLife v6.13.3 | Open Movement GUI v1.0.0.30 | PALconnect v8.9.8.48 & PALbatch v8.9.4.17 |

**Section B**

Bland-Altman agreement between Actigraph Gt3x and Axivity AX3 estimates of physical behaviors are presented in figure 1(a) & figure 1(b). Differences are based on free-living measurements; therefore, data from 19 participants is included. The following physical activities and postures are depicted: B1 = Lie/Sit; B2 = Stand; B3 = Move; B4 = walk; B5 = run; B6 = stair climbing; B7 = cycle; B8 = stepping.

Figure 1(a) **–** Bland-Altman agreements between Actigraph Gt3x and Axivity AX3 physical behavior estimates


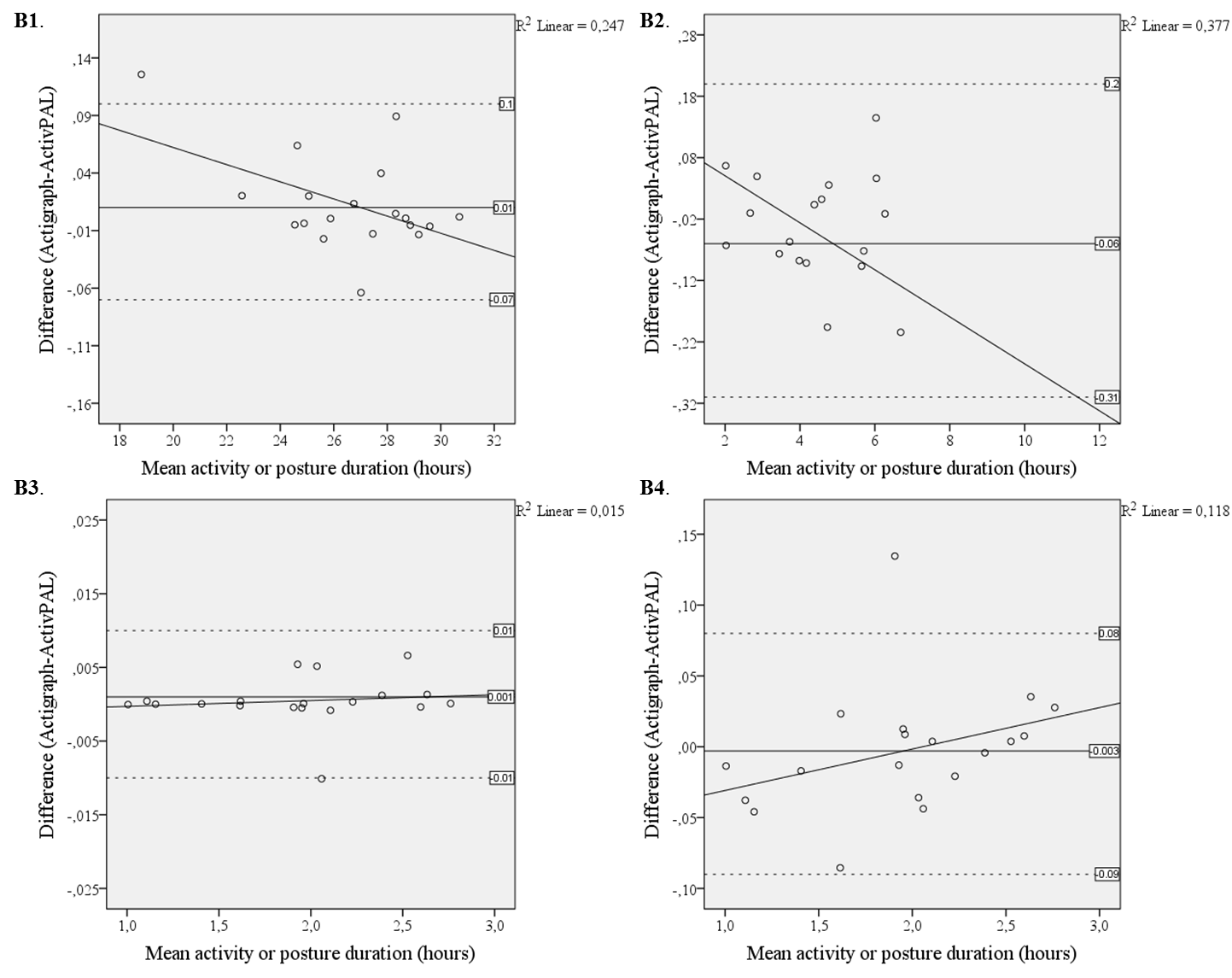


Y-axis reference lines from indicate mean, upper and lower limit of agreement (±1.96 SD).

Figure 1(b) **–** Bland-Altman agreement between Actigraph Gt3x and Axivity AX3 physical behavior estimates


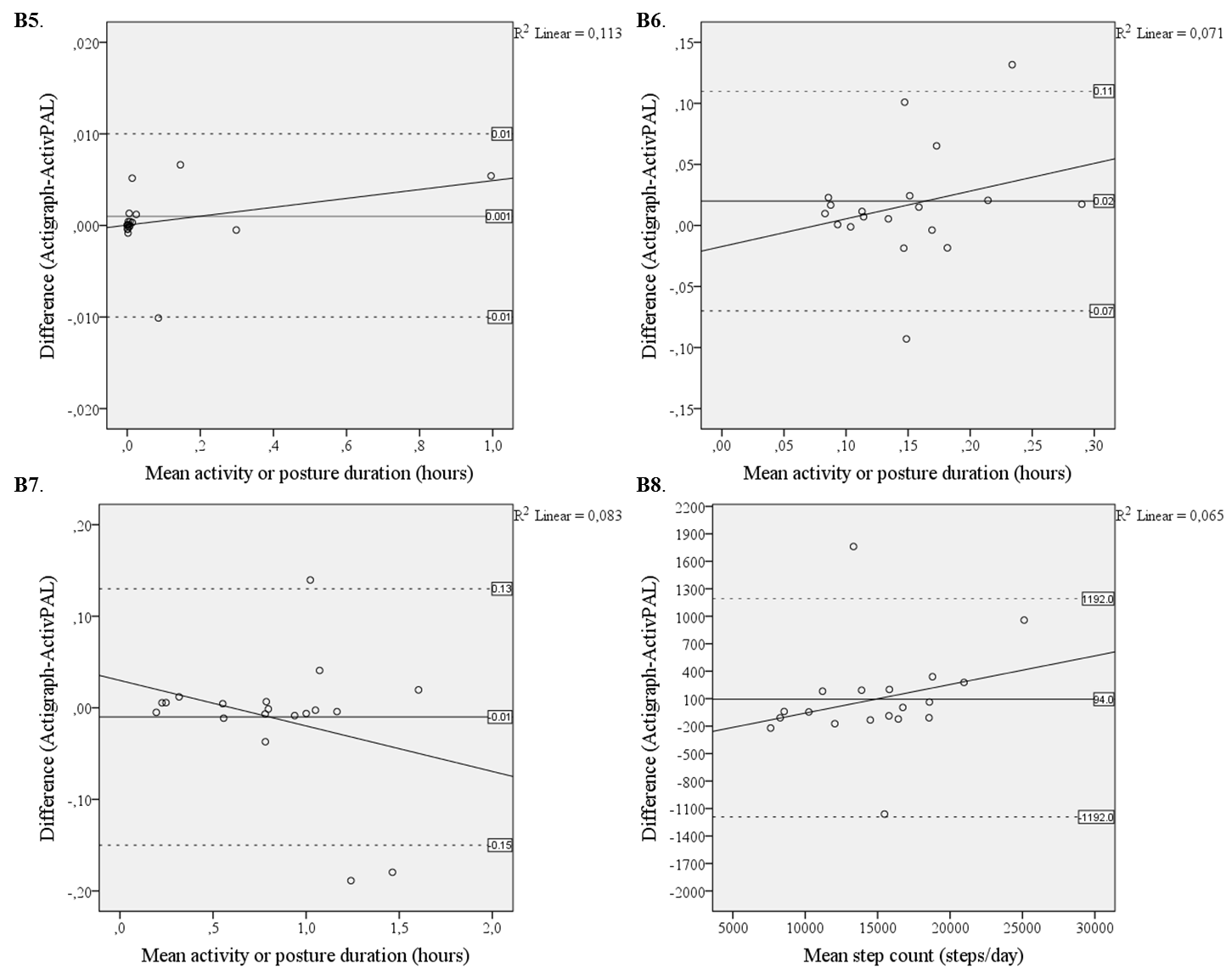


Y-axis reference lines from indicate mean, upper and lower limit of agreement (±1.96 SD).

**Section C**

Bland-Altman agreement between Actigraph Gt3x and ActivPAL Micro4 estimates of physical behaviors are presented in figure 2(a) & figure 2(b). Differences are based on free-living measurements; therefore, data from 19 participants is included. The following physical activities and postures are depicted: C1 = Lie/Sit; C2 = Stand; C3 = Move; C4 = walk; C5 = run; C6 = stair climbing; C7 = cycle; C8 = stepping.

Figure 2(a) **–** Bland-Altman agreement between Actigraph Gt3x and ActivPAL Micro4 estimates of physical behavior estimates


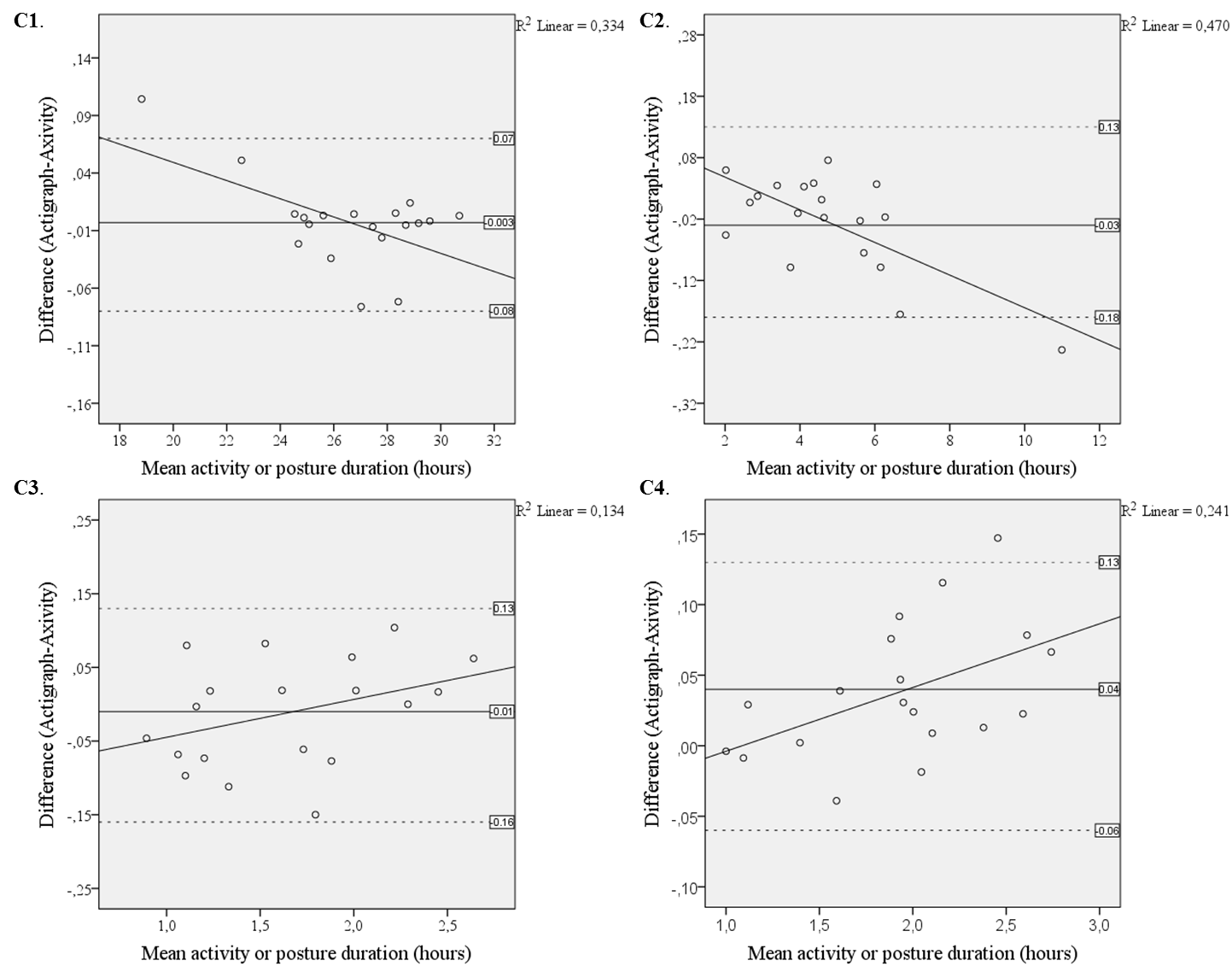


Y-axis reference lines from indicate mean, upper and lower limit of agreement (±1.96 SD).

Figure 2(b) – Bland-Altman agreement between Actigraph Gt3x and ActivPAL Micro4 physical behavior estimates


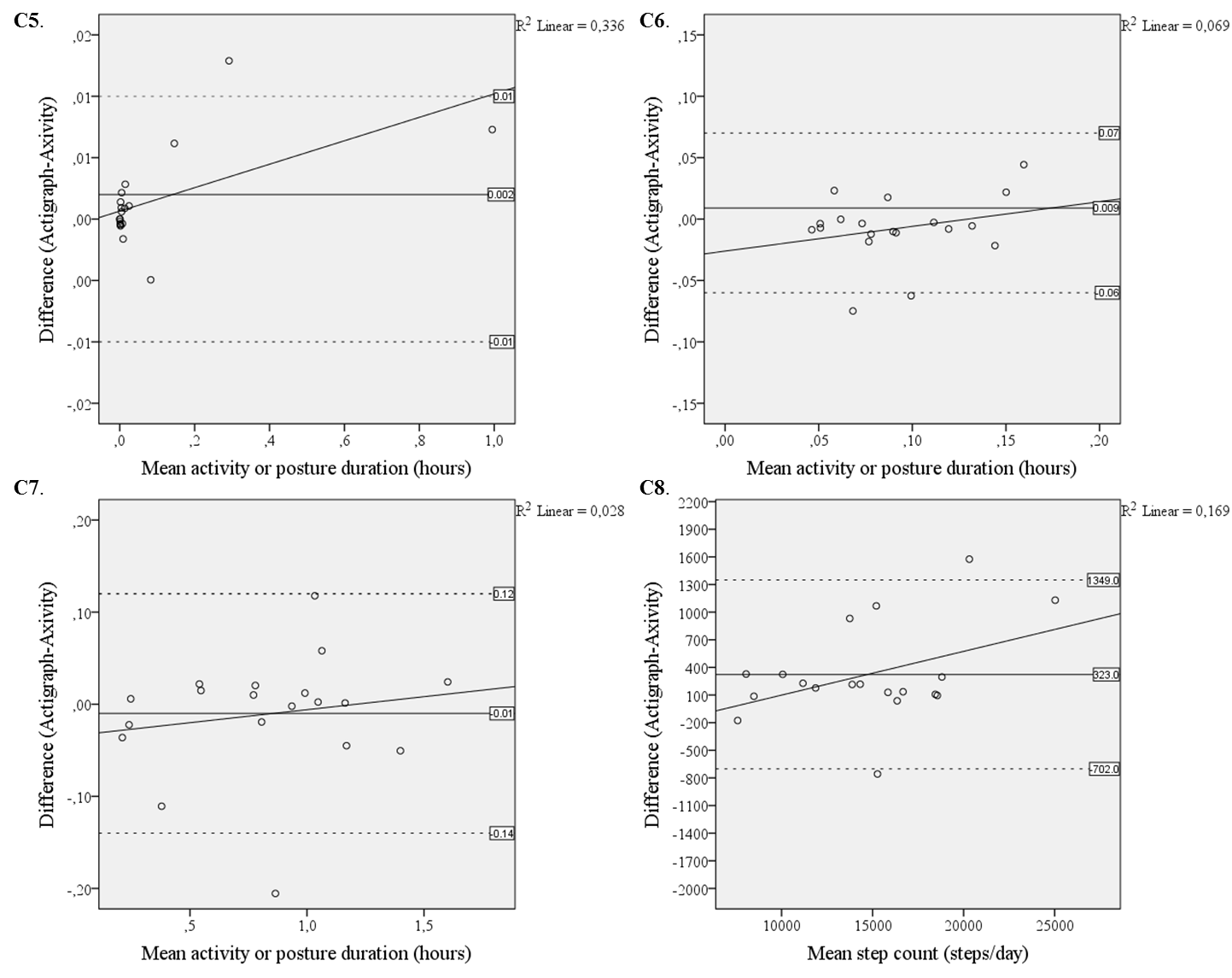


Y-axis reference lines from indicate mean, upper and lower limit of agreement (±1.96 SD).

**Section D**

Bland-Altman agreement between Axivity AX3 and ActivPAL Micro4 estimates of physical behaviors are presented in figure 2(a) & figure 2(b). Differences are based on free-living measurements; therefore, data from 19 participants is included. The following physical activities and postures are depicted: D1 = Lie/Sit; D2 = Stand; D3 = Move; D4 = walk; D5 = run; D6 = stair climbing; D7 = cycle; D8 = stepping.

Figure 3(a) – Bland-Altman agreement between Axivity AX3 and ActivPAL Micro4 physical behavior estimates

**
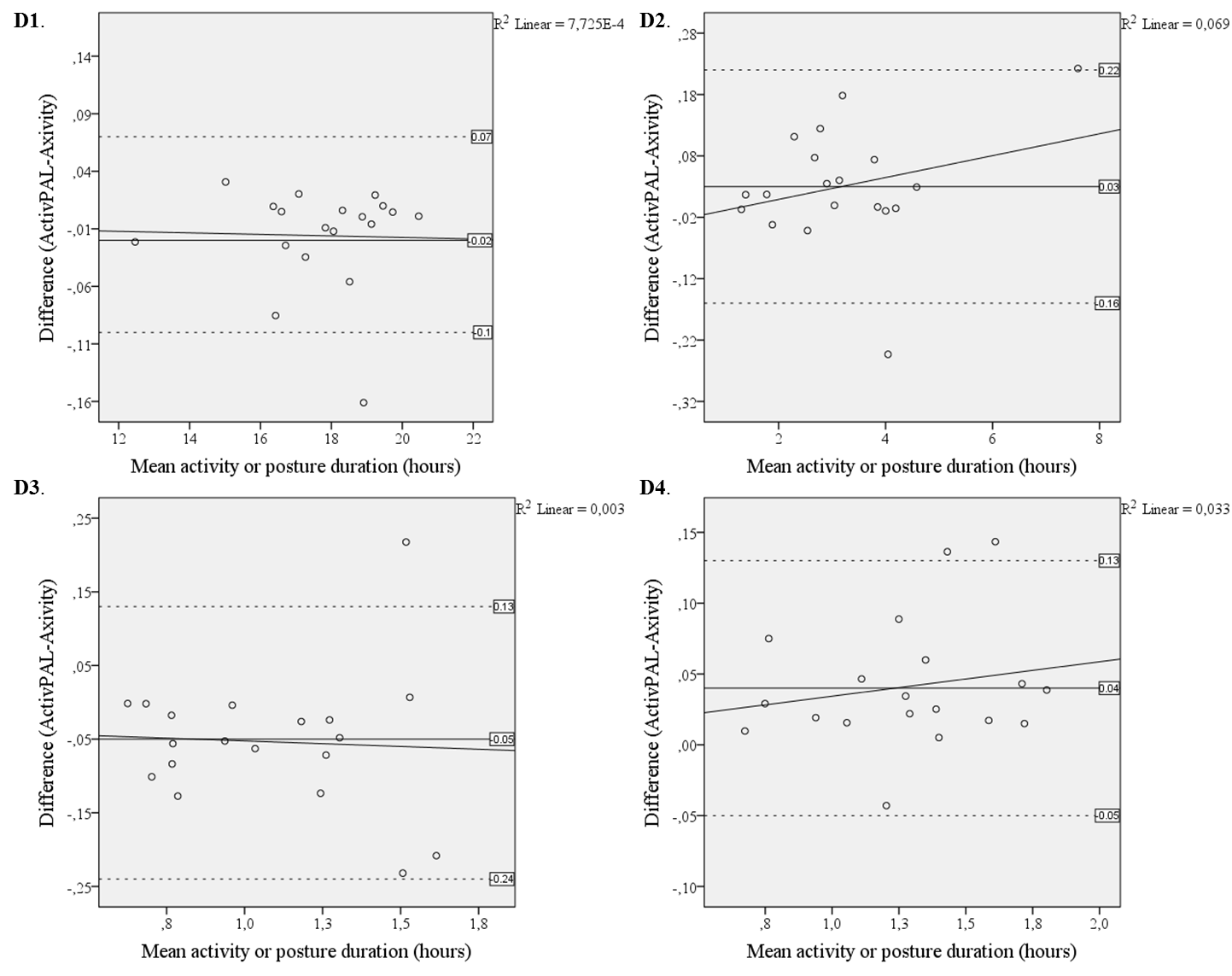
**

Y-axis reference lines from indicate mean, upper and lower limit of agreement (±1.96 SD).

Figure 3(b) – Bland-Altman agreement between Axivity AX3 and ActivPAL Micro4 physical behavior estimates

**
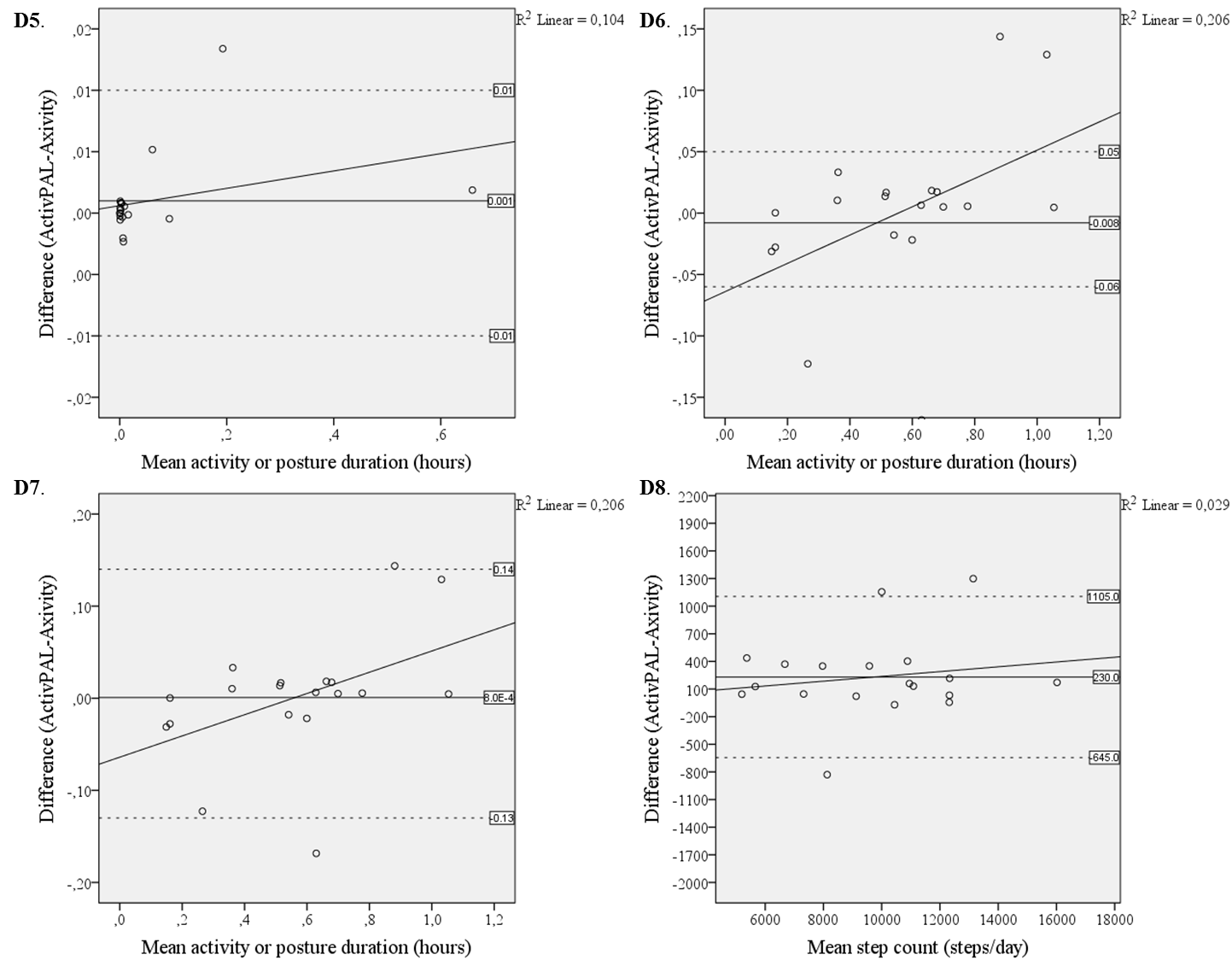
**

Y-axis reference lines from indicate mean, upper and lower limit of agreement (±1.96 SD).

**Section E**

Operational definitions used for the annotation of video observations recorded during physical behavior measurement in the semi-standardized setting. To restrict judgments to the current frame and reduce the tendency to predict movements, the following conditions were pre-defined for annotating: *“The judgement of a posture or activity may only take the current frame (n) and the previous frame (n-1) into account. Therefore to change classification frame (n) has to distinctly fit a new classification from frame (n-1).”* Where (n) is the frame number. Definitions were grouped into four categories as documented in table E.

Table E – Definitions used in the annotation of video observations

| Category | Activity | Definition |
| --- | --- | --- |
| Upright activities and postures – the participant is on their feet, with a thigh angle above 45 degrees with respect to the ground | Standing | No movement of the center of gravity outside of the base of support |
|  | Move | Movement of the center of gravity outside the base of support, while not classified as any other activity |
|  | Walking | Movement of the center of gravity in the anterior direction. |
|  | Stair climbing | Walking while on a stair case, including between stair platforms, not including static standing. |
|  | Running | Movement of the center of gravity in the anterior direction, with both feet leaving the ground simultaneously. |
| Posture-based classification | Sitting | A trunk posture of over a 45 degree angle form the ground, with a thigh angle below 45 degrees also relative to the ground. Buttocks should be the primary weight bearing body part. |
| Other activities | Cycling | Once the participants weight is fully supported by the bicycle |
| Miscellaneous | Invisible | Participant is obstructed and therefore activity in not classifiable on frame |
|  | Other | Observed activities not fitting into the definitions above |
